# NOVEL BEHAVIORAL HEALTH APPROACHES FOR OLDER ADULTS IN UNDERSERVED COMMUNITIES

**DOI:** 10.1093/geroni/igad104.1798

**Published:** 2023-12-21

**Authors:** Ryan Mace, Ana-Maria Vranceanu, Kathi Heffner

## Abstract

Chronic conditions and related stressors among older adults are leading drivers of disability, morbidity, and healthcare costs in the US. Underserved communities have the highest rates of chronic conditions and are at the greatest risk for negative health outcomes. Novel behavioral health approaches are needed to address barriers faced by these communities (e.g., stigma, discrimination, structural inequalities) that exacerbate health disparities. Consistent with the GSA 2023 theme, this symposium will showcase multidisciplinary initiatives to build bridges to clinical care, catalyze community-engaged research, and empower underserved older adults. Presenter 1 will present the protocol, preliminary results, and “lessons learned” recruiting ethnoracially diverse older adults for a fully-powered efficacy RCT (target N=260) of Active Brains, a virtual group mind-body walking program aided by Fitbit targeting comorbid chronic pain and early cognitive decline. Presenter 2 will present focus groups with patient and medical provider stakeholders to culturally adapt a mind-body activity program (GetActive) for disadvantaged older adults with chronic pain in community clinics. Presenter 3 will present the rationale, design, and challenges of an RCT testing the effectiveness of a peer support program (Peer Enhanced Depression Care) to alleviate depressive symptoms in ethnoracially diverse and/or low-income older adults (N=160). Presenter 4 will present a stakeholder-partnered approach to equitable home care for older adults and caregivers in diverse communities by eliminating transportation-related barriers. The Discussant will contextualize these initiatives in the broader field of gerontology and share research from their Roybal Center to engage community agencies and ethnoracially diverse older adults.

